# Triglyceride glucose-body mass index as a novel predictor of slow coronary flow phenomenon in patients with ischemia and nonobstructive coronary arteries (INOCA)

**DOI:** 10.1186/s12872-024-03722-4

**Published:** 2024-01-19

**Authors:** Zhi-peng Li, Juan Chen, Qi Xin, Xiao-yang Pei, Hong-li Wu, Zhi-xu Tan

**Affiliations:** 1 Department of Cardiology, The Affiliated Hospital of Inner Mongolia Minzu University, Tongliao, China; 2 Department of Cardiology, Shenzhen Luohu Hospital Group Luohu People’s Hospital (The Third Affiliated Hospital of Shenzhen University), Shenzhen, China

**Keywords:** Triglyceride glucose-body mass index, Slow coronary flow fhenomenon, Ischemia and nonobstructive coronary arteries, Predictors

## Abstract

**Background:**

The triglyceride glucose-body mass index (TyG-BMI index) has been suggested as a novel predictor of insulin resistance. However, its predictive value for slow coronary flow phenomenon (SCFP) in patients with ischemia and nonobstructive coronary arteries (INOCA) remains unclear.

**Methods:**

We consecutively recruited 1625 patients with INOCA from February 2019 to February 2023 and divided them into two groups based on thrombolysis in myocardial infarction (TIMI) frame counts (TFCs): the SCFP group (*n* = 79) and the control group. A 1:2 age-matched case–control study was then performed. The TyG-BMI index was calculated as ln [plasma triglyceride (mg/dL) × fasting blood glucose (mg/dL)/2] × BMI.

**Results:**

TyG-BMI index in the SCFP group (218.3 ± 25.2 vs 201.0 ± 26.5, *P* < .001) was significantly higher than in the normal controls. TyG-BMI index also increased with the number of coronary arteries involved in the SCFP. Multivariate logistic regression analysis showed that TyG-BMI, BMI, and TG were independent predictors for SCFP. Receiver operating characteristic (ROC) curve analysis showed that when the TyG-BMI index was above 206.7, the sensitivity and specificity were 88.6% and 68.5%, respectively, with an AUC of 0.809 (95% CI: 0.756–0.863, *P* = .027). Combined BMI with TG, the TyG-BMI index had a better predictive value for SCFP than BMI and TG (*P* < .001).

**Conclusion:**

The TyG-BMI index was an independent predictor for SCFP in INOCA patients, and it had a better predictive value than BMI and TG.

## Introduction

Ischemia with nonobstructive coronary arteries (INOCA) often occurs in patients with angina and normal coronary arteries, confirmed by angiography [1]. Some of these patients are diagnosed with slow coronary flow phenomenon (SCFP), which is defined as slow blood flow in the main coronary arteries despite the absence of significant coronary stenosis (< 40%). The coronary flow of blood is determined by the thrombolysis in myocardial infarction (TIMI) frame count (TFC) during diagnostic coronary angiography (CAG) [2]. The incidence of SCFP in patients with INOCA ranges from 1 to 7% [3]. SCFP is associated with an increased risk of adverse cardiovascular events, and up to 80% of SCFP patients experience recurrent angina attacks at rest. Approximately 20% of SCFP patients are readmitted to the emergency room or coronary care unit [4]. The recurrent episodes of angina and the fear of sudden death significantly diminish the quality of life for individuals with SCFP. However, the underlying cause of SCFP remains unclear.

The triglyceride glucose index (TyG index), which combines fasting triglycerides with fasting blood glucose levels, has been proposed as a reliable surrogate marker for insulin resistance [5]. Previous studies have shown a close relationship between the TyG index and arterial stiffness, as assessed by brachial-ankle pulse wave velocity [6]. Cardiovascular conditions are characterized with increased burden of inflammation. Body mass index (BMI) is an important clinical indicator used to assess obesity and insulin resistance [7], as well as chronic inflammation [8]. Similarly, triglyceride-based indexes are considered metabolic and low-grade inflammatory markers in cardiovascular disease [9]. The TyG-BMI index, which considers both the TyG index and BMI, has been suggested as a surrogate marker for insulin resistance in Chinese nondiabetic individuals [10]. Given that SCFP is closely associated with inflammation [11], insulin resistance [12], and arterial stiffness [13], we hypothesized that the TyG-BMI index may be involved in the pathophysiological processes of SCFP. Therefore, the aim of this study is to investigate the correlation between the TyG-BMI index and SCFP.

## Methods

### Study population

**Fig. 1:**
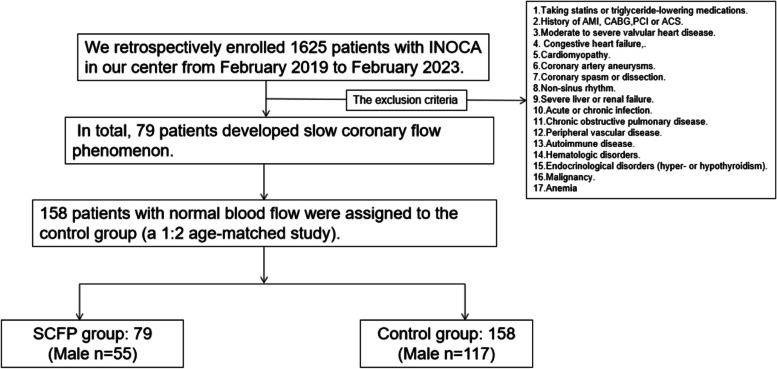
The study flowchart. INOCA, ischemia and nonobstructive coronary arteries; AMI, acute myocardial infarction; CABG, coronary artery bypass grafting; ACS,acute coronary syndrome; SCFP, slow coronary flow phenomenon

This is a retrospective observational study with a relatively small sample size. From February 2019 to February 2023, we consecutively enrolled 1625 patients with INOCA. These patients were then divided into two groups based on their TFCs. In total, 79 patients developed slow coronary flow phenomenon (SCFP) and were assigned to the SCFP group, while 158 patients with normal blood flow were assigned to the control group (a 1:2 age-matched). The exclusion criteria were as follows: taking statins or triglyceride-lowering medications, previous history of myocardial infarction, coronary artery bypass grafting or PCI, acute coronary syndrome, moderate to severe valvular heart disease, congestive heart failure, cardiomyopathy, coronary artery aneurysms, coronary spasm or dissection, non-sinus rhythm, severe liver or renal failure, acute or chronic infection, chronic obstructive pulmonary disease, peripheral vascular disease, autoimmune disease, hematologic disorders, endocrinological disorders (hyper- or hypothyroidism), malignancy, and anemia (hemoglobin level < 12 g/dL for women or < 13 g/dL for men) according to the World Health Organization criteria) [11] (Fig. 1).

### Laboratory measurements and definitions

The routine laboratory parameters, including fasting glucose, creatinine, uric acid, total cholesterol (TC), triglyceride (TG), high-density lipoprotein cholesterol (HDL-C), and low-density lipoprotein cholesterol (LDL-C) were tested using fasting blood samples after a night of fasting. All the plasma samples were assessed using an auto-analyzer (Hitachi 747, Tokyo, Japan) at the central laboratory in our hospital. Serum TC, TG, LDL-C, and HDL-C levels were measured with the enzymatic colorimetric method. The fasting glucose levels were measured using the hexokinase/glucose-6-phosphate dehydrogenase method with the coefficient of variation using blind quality control specimens < 2.0%.The TyG-BMI index was calculated as ln [plasma triglyceride (mg/dL) × fasting blood glucose (mg/dL) / 2] × BMI [5].

### Coronary angiography

Coronary angiography was performed using the standard Judkins technique with 30 frames per second (fps). The preferred access of the procedure was the right radial artery. The coronary blood flow was calculated using TFC [2] by two interventional cardiologists [2]. The first frame was defined as > 70% lumen filling with an antegrade contrast agent, and the last frame was determined as the antegrade contrast agent filling to a certain distal landmark for different coronary arteries. The distal bifurcation (“whale’s tail”), distal bifurcation of the obtuse marginal branch, and the first branch of the posterolateral artery were used as the distal landmark for the left anterior descending artery (LAD), left circumflex artery (LCX), right coronary artery (RCA), respectively. The TFCs for the LAD divided by 1.7 yield the corrected TFCs (cTFCs) [14]. The TFCs for normal epicardial coronary arteries were 36.2 ± 2.6 for the LAD (21.1 ± 1.5 cTFC for LAD), 22.2 ± 4.1 for the LCX, and 20.4 ± 3 for the RCA [14]. Any patients with higher TFCs than these threshold values were considered SCFP.

### Statistical analysis

The SPSS 22.0 was used for data analysis. Categorical variables were expressed as rates or percentages, which were compared using chi-square or the Fisher exact test. Shapiro–Wilk test was used for normality analysis in continuous variables. The continuous variables were displayed as the mean ± standard deviation or median, as applicable, which were compared using the one-way ANOVA test or the Kruskal–Wallis test. The univariable regression analysis was used to assess the potential risk factors associated with SCFP, and logistic regression analysis was used to investigate the independent predictors of SCFP. Receiver operating characteristic (ROC) curve analysis was generated to assess the predictive ability of the risk factors for SCFP. A 2-sided *P* < 0.05 was considered statistically significant.

## Results

### Baseline and clinical characteristics

The demographic characteristics, cardiovascular disease comorbidities, and medication history are displayed in Table 1. There were no significant differences in the gender distributions and the proportion of smokers between the two groups (*P* > 0.05). The heart rate, systolic blood pressure, diastolic pressure, and medications were also comparable between the two groups (*P* > 0.05). However, the SCFP patients had a higher proportion of dyslipidemia, hypertension, and diabetes mellitus (*P* < 0.05) (Table 1).

**Table 1:** Baseline characteristics and medication of the two groups

	SCFP group (*n* = 79)	Control group (*n* = 158)	*P* value
Age, years	61.1 ± 9.0	61.1 ± 9.0	1
Male sex, n (%)	55(69.6)	117(74.1)	0.537
BMI	24.6 ± 2.4	23.4 ± 2.8	0.001
Heart rate	72.1 ± 11.2	72.6 ± 10.8	0.752
Systolic blood pressure, mmHg	124.5 ± 18.2	123.7 ± 13.1	0.691
Diastolic pressure, mmHg	76.3 ± 11.4	75.4 ± 8.7	0.488
Current smoking, n (%)	20(25.3)	49(31.0)	0.448
Dyslipidemia, n (%)	29(36.7)	33(20.9)	0.012
Hypertension, n (%)	46(58.2)	64(40.5)	0.013
Diabetes mellitus, n (%)	23(29.1)	26(16.5)	0.028
hyperuricemia, n (%)	16(20.3)	25(15.8)	0.467
ACEI/ARB/ARNI, n (%)	18(22.8)	32(20.3)	0.736
Beta-blocker, n (%)	15(19.0)	28(17.7)	0.859
Calcium canal blocker, n (%)	22(27.8)	31(19.6)	0.186
Antiplatelet, n (%)	16(20.3)	34(21.5)	0.867
Statin, n (%)	23(29.1)	48(30.4)	0.881
Nitrates, n (%)	24(30.4)	40(25.3)	0.440

*BMI* body mass index, *ACEI* angiotensin-converting enzyme inhibitor, *ARB* angiotensin II receptor blocker, *ARNI* angiotensin receptor enkephalinase inhibitor

### Laboratory parameters of the two groups

**Fig. 2:**
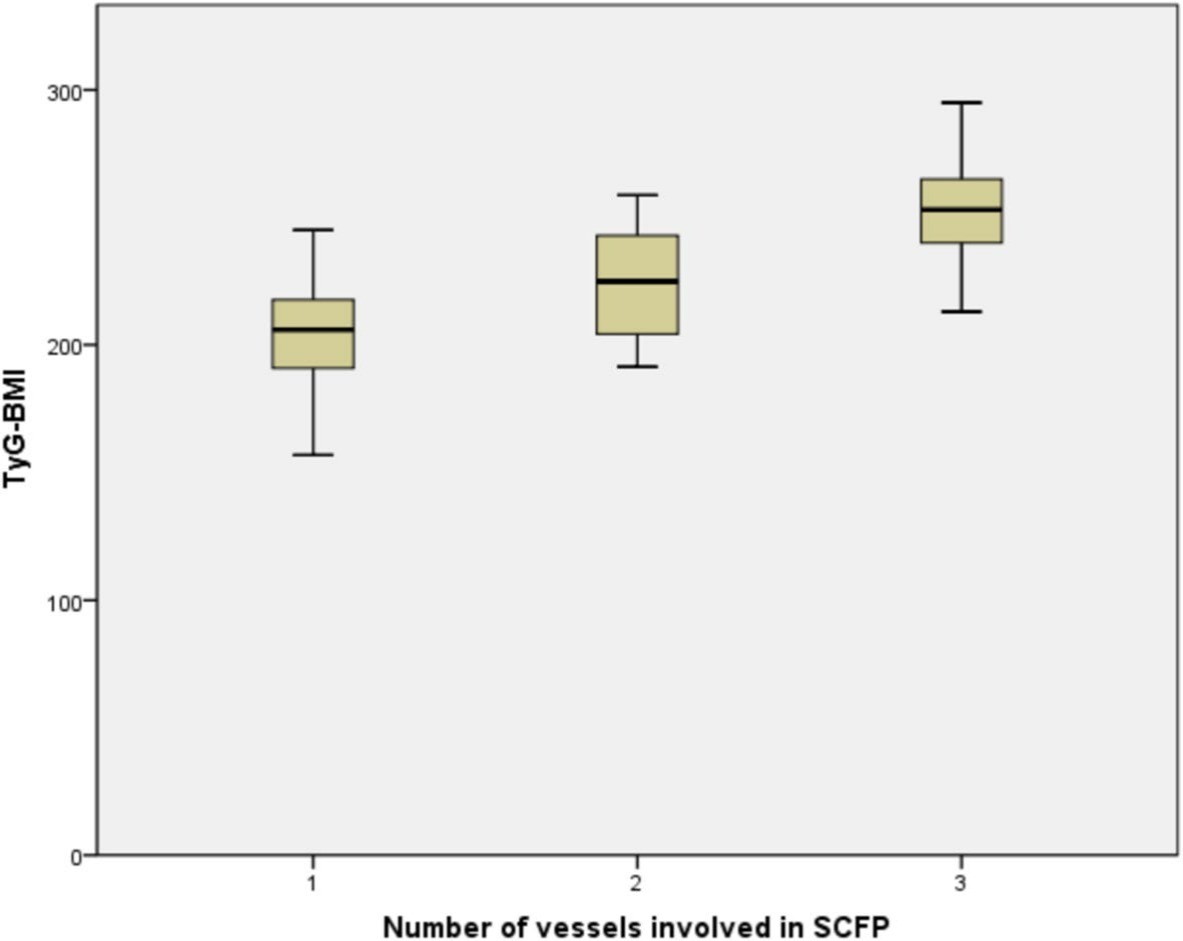
Relationship between the number of vessels involved in SCFP and TyG-BMI. TyG-BMI, triglyceride glucose body mass index; SCFP, slow coronary flow phenomenon

The laboratory parameters are presented in Table 2. There were no statistically significant differences in serum creatinine, uric acid, and high-density lipoprotein cholesterol levels between the SCFP patients and the controls. However, the SCFP patients showed significantly higher levels of fasting glucose (6.1 ± 1.9 vs 5.4 ± 1.8, *P* = 0.005), total cholesterol (4.4 ± 1.2 vs 4.1 ± 1.0, *P* = 0.026), triglyceride (1.6 ± 0.5 vs 1.4 ± 0.6, *P* = 0.015), low-density lipoprotein cholesterol (2.8 ± 0.0 vs 2.5 ± 0.9, *P* = 0.013), and TyG-BMI index (218.3 ± 25.2 vs 201.0 ± 26.5, *P* < 0.001) compared to the controls (Table 2). Furthermore, our investigation revealed that the TyG-BMI index increased with the number of coronary arteries involved in SCFP (Fig. 2).

**Table 2:** Laboratory parameters of the two groups

	SCFP group (*n* = 79)	Control group (*n* = 158)	*P* value
Fasting glucose, mmol/l	6.1 ± 1.9	5.4 ± 1.8	0.005
Cr, mmol/l	73.0 ± 25.3	73.9 ± 22.6	0.767
Uric acid, umol/L	318.7 ± 70.8	305.5 ± 80.1	0.301
Total cholesterol, mmol/l	4.4 ± 1.2	4.1 ± 1.0	0.026
Triglyceride, mmol/l	1.6 ± 0.5	1.4 ± 0.6	0.015
HDL-C, mmol/l	1.0 ± 0.3	1.1 ± 0.3	0.363
LDL-C, mmol/l	2.8 ± 1.0	2.5 ± 0.9	0.013
TyG-BMI	218.3 ± 25.2	201.0 ± 26.5	< 0.001

*HDL-C* high-density lipoprotein cholesterol, *LDL-C* low-density lipoprotein cholesterol, *TyG-BMI* triglyceride glucose-body mass index

### Angiographic characteristics of the two groups

The angiographic characteristics of patients with SCFP are displayed in Table 3. The TFCs of the three coronary arteries were significantly higher in SCFP patients compared with the controls (*P* < 0.001). In terms of the vessels involved in SCFP, the RCA (68.4%) was the most frequently affected, followed by LAD (59.5%), and LCX the last (58.2%). In patients with SCFP, a single coronary artery affected (40.5%) was the most common phenomenon, followed by two arteries (32.9%) and the last three coronary arteries (26.6%) (Table 3).

**Table 3:** Angiographic characteristics of the two groups

	SCFP group (*n* = 79)	Control group (*n* = 158)	*P* value
TIMI frame count			< 0.001
LAD	26.3 ± 4.3	20.9 ± 1.8	
LCX	24.2 ± 4.4	19.0 ± 3.8	
RCA	26.4 ± 5.1	18.9 ± 2.1	
mean TFC	25.6 ± 12.9	19.6 ± 5.9	
Distribution of SCFP			
LAD, n (%)	47(59.5)		
LCX, n (%)	46(58.2)		
RCA, n (%)	54(68.4)		
Numbers of vessels involved in SCFP			
1, n (%)	32(40.5)		
2, n (%)	26(32.9)		
3, n (%)	21(26.6)		

*TIMI* thrombolysis in myocardial infarction, *LAD* left anterior descending artery, *LCX* left circumflex artery, *RCA* right coronary artery, *TFC* TIMI frame count, *SCFP* slow coronary flow fhenomenon

### Predictors of SCFP

**Fig. 3:**
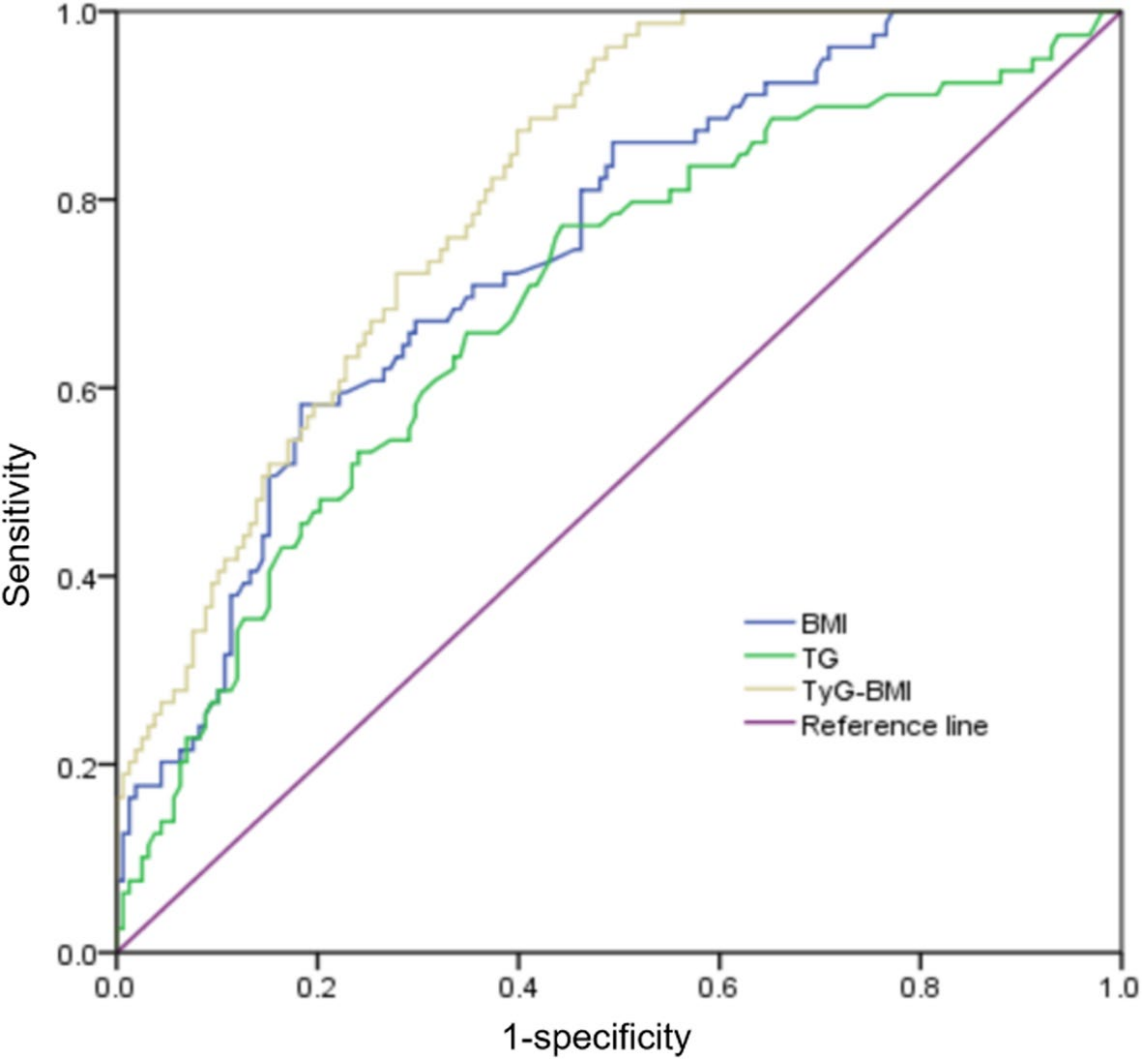
ROC curve showing the predicting value of risk factors for the presence of SCFP. BMI, body mass index; TG, triglyceride; TyG, triglyceride glucose index; SCFP, slow coronary flow phenomenon

Univariate analysis showed that SCFP was associated with BMI, Dyslipidemia, hypertension, diabetes mellitus, fasting glucose, total cholesterol, triglyceride, LDL-C, and TyG-BMI index/10 (*P* < 0.05). Multivariate logistic regression analysis revealed that BMI, triglyceride, and TyG-BMI index/10 were independent predictors for SCFP(Table 4). The ROC curve showed that when BMI was more than 25.5, the sensitivity and specificity were 64.1% and 81.6%, respectively, and the area under the ROC curve (AUC) was 0.748 (95% CI: 0.648–0.811, *P* = 0.033). When the TG level was more than 1.4, the sensitivity and specificity were 77.2% and 65.8%, respectively, and the AUC was 0.693 (95% CI: 0.621–0.764, *P* < 0.001). When the TyG-BMI index level was more than 206.7, the sensitivity and specificity were 88.6% and 68.5%, respectively, and the AUC was 0.809 (95% CI: 0.756–0.863, *P* = 0.027) (Table 5). The combination of BMI and triglyceride, the TyG-BMI index, showed a better predictive value for SCFP compared to BMI and triglyceride alone (*p* < 0.001) (Fig. 3).

**Table 4:** Univariate and multivariate logistic regression analysis for presence of SCFP

	Univariate analysis			Multivariate analysis		
	OR	95% CI	P值	OR	95% CI	P值
BMI	1.121	1.015–3.369	0.028	1.118	1.011–3.391	**0.033**
Dyslipidemia	1.320	1.012–5.321	0.025	1.335	0.892–6.124	0.512
Hypertension	1.391	1.081–4.213	0.037	1.353	0.798–4.332	0.429
Diabetes mellitus	1.521	1.069–3.528	0.027	1.426	0.802–4.015	0.461
Fasting glucose	1.624	1.092–3.568	0.026	1.582	0.821–3.602	0.380
Total cholesterol	2.021	1.123–8.231	0.035	1.995	0.726–8.059	0.624
Triglyceride	2.465	1.109–7.265	0.019	2.424	1.117–8.021	0.030
LDL-C	1.925	1.039–5.321	0.029	1.902	0.914–5.394	0.295
TyG-BMI/10	1.526	1.017–8.022	0.024	1.582	1.064–8.204	0.019

*BMI* body mass index, *LDL-C* low-density lipoprotein cholesterol, *TyG-BMI* triglyceride glucose-body mass index

**Table 5:** ROC analysis of BMI, TG and TyG-BMI

	AUC	95%CI	sensitivity	specificity	Positive predictive values	LR +	Negative predictive values	LR-	Youden index
BMI	0.748	0.648–0.811	64.1%	81.6%	64.1%	4.434	81.6%	0.225	0.457
TG	0.693	0.621–0.764	77.2%	65.8%	77.2%	1.924	65.8%	0.520	0.430
TyG-BMI	0.809	0.756–0.863	88.6%	68.5%	88.6%	2.175	68.5%	0.460	0.544

*BMI* body mass index, *LDL-C* low-density lipoprotein cholesterol, *TyG-BMI* triglyceride glucose-body mass index

## Discussion

In this study, we observed that the TyG-BMI index was significantly higher in SCFP patients compared to the controls. Furthermore, multivariate logistic regression analysis revealed that the TyG-BMI index was an independent predictor of SCFP in INOCA patients. Additionally, we found that the TyG-BMI index increased with the number of coronary arteries involved in SCFP. To our knowledge, this is the first study to investigate the association between the TyG-BMI index and SCFP in patients with INOCA.

SCFP had been suggested as a common angiographical finding in patients with INOAC. The prevalence of SCFP varies from 1 to 7%. Similar to previous studies, our study found that the incidence of SCFP was 4.8%. We also discovered that from the aspect of the vessels involved in the SCFP, the RCA was the most frequently affected, then LAD and the last was LCX. In patients with SCFP, a single coronary artery affected was the most common phenomenon, followed by two arteries; the last was three coronary arteries. A previous study suggested that 80% of patients with SCFP experienced recurrent angina attacks at rest. Moreover, 20% of the SCFP patients were readmitted to emergency room or coronary care unit [4]. SCFP may associated with traditional cardiovascular risk factors, such as hypertension, diabetes mellitus, dyslipidemia, and hyperuricemia. However, unfortunately, the literature has not consistently demonstrated the risk factors of SCFP. This study found that SCFP was related to BMI, hypertension, diabetes mellitus, and dyslipidemia. However, multivariate logistic regression analysis revealed that these traditional cardiovascular risk factors did not correlate with SCFP. So we speculated that different from previous classical cardiovascular diseases, SCFP may have a specific pathophysiology [15, 16], and therefore, investigating the predictive factors of SCFP was important to clinical practice.

The pathophysiology of SCFP remains unclear. However, more and more studies indicated that SCFP was associated with insulin resistance [12], subclinical atherosclerosis [13] and endothelial dysfunction [4] and chronic inflammation [11, 17]. Ozcan T et al. discovered an association between TFCs and increased insulin resistance, as assessed using the homeostasis model assessment index. They also proposed that insulin resistance may play a role in the development of SCFP [12]. Camsari et al. discovered diffuse intimal thickening of coronary arteries in SCFP [18]. Another study revealed that carotid intima-media thickness was closely correlated with TFC in SCFP patients [19]. Wang Y et al. discovered that as a reliable biomarker of endothelial function, plasma thrombomodulin levels were significantly higher in SCFP patients than in the controls and were also associated with TFCs in the individuals included [4]. Dai XT discovered that as a novel indicator of chronic inflammation, the systemic immune-inflammation index was associated with SCFP and was an independent predictor for SCFP [11].

TyG index has been suggested as a reliable surrogate biomarker for insulin resistance [5]. Insulin resistance has been suggested as a risk factor for both the progress of type 2 diabetes mellitus and cardiovascular diseases [20, 21]. A higher TyG index was associated with poor outcomes in acute coronary syndrome undergoing PCI [22], the severity of coronary artery disease [23], atrial fibrillation [24], and repeat revascularization and in-stent restenosis in patients with chronic coronary syndrome undergoing PCI [25]. Moreover, an elevated level of TyG index was an independent predictor for increased arterial stiffness [26], increased odds of atherosclerosis in coronary arteries [27], and increased carotid atherosclerosis [28]. Yuksel Y et al. suggested that the TyG index was an independent predictor for SCFP [29]. Insulin resistance may also decrease the release of nitric oxide and increase the overproduction of reactive oxidative stress, which could damage endothelial function [30]. Furthermore, insulin resistance may lead to an increased release of tissue factors, which is associated with inflammation [31]. Moreover, a higher BMI indicates obesity, insulin resistance [32], and inflammation [8]. Therefore, the TyG-BMI index, consisting of three classic cardiovascular disease risk markers, lipid-related, glucose-related, and obesity-related factors, has been suggested to be a reliable predictor of insulin resistance [32]. The TyG-BMI index has also been suggested to be closely associated with traditional cardiovascular risk factors, including hypertension and diabetes [32]. Since SCFP was closely related to insulin resistance [11], subclinical atherosclerosis [13], endothelial dysfunction [4], and BMI [33], we speculated that the TyG-BMI index could play a role in the pathophysiology of SCFP. Combined with the TyG index and BMI, the TyG-BMI index may have a better predictive value for SCFP. We discovered that patients with SCFP had a higher level of TyG-BMI index, and the TyG-BMI index increased as the number of coronary arteries involved in the SCFP. Multivariate logistic regression analysis revealed that the TyG-BMI index was an independent predictor for SCFP. We speculated that the mechanisms associating TyG-BMI with SCFP are as follows: (1) insulin resistance plays the main mechanism; (2) oxidative stress, chronic inflammatory reaction, and endothelial dysfunction; and (3) coronary or systemic atherosclerosis relating to various risk factors and endothelial dysfunction.

SCFP is quite a complex phenomenon with many factors unclear, including the risk factors, the pathophysiology, and the prognosis. TyG-BMI index consists of three clinical factors, including lipid-related, glucose-related, and obesity-related factors, and better reflects insulin resistance, endothelial dysfunction, oxidative stress status, and coronary atherosclerosis. We conclude that the TyG-BMI index could improve the predictive value of SCFP compared with BMI or TyG alone. The TyG-BMI index was easily acquired and may be a promising parameter for predicting SCFP in INOCA.

### Limitations

However, this study also had some limitations. First, this was a single-center study with a small sample size. Secondly, since the risk factors of SCFP remain unclear, despite our efforts to include additional factors, there may still be some residual covariates that could potentially affect the predictive value of the TyG-BMI index. Thirdly, the individuals included represented a specific population, which could not extend to other clinical circumstances, such as acute coronary syndrome with INOCA. Finally, large-scale, multicenter studies are needed to verify our conclusion.

### Future directions

In clinical practice, the TyG-BMI index could be used as an indicator for the prediction of SCFP. The TyG-BMI index could be a promising treatment target from the therapeutic perspective. So, we speculate that weight loss, statin, and aggressive blood glucose-lowering strategies could be promising and appropriate treatments for patients with SCFP. However, large, multicenter randomized controlled trials are needed to confirm these speculations.

## Conclusion

TyG-BMI index was an independent predictor for SCFP in patients with INOCA. The TyG-BMI index could significantly improve the predictive value of SCFP compared to BMI and TyG alone.

## Data Availability

The datasets generated and analysed during the current study are not publicly available due to a further study of this area but are available from the corresponding author on reasonable request.

## Abbreviations

SCFP: Slow coronary flow phenomenon
INOCA: Ischemia and nonobstructive coronary arteries
BMI: Body mass index
TyG index: Triglyceride glucose index
TyG-BMI index: Ctriglyceride glucose-body mass index
TIMI: Thrombolysis in Myocardial Infarction
TFC: TIMI frame count
LAD: Left anterior descending
LCX: Left circumflex
RCA: Right coronary artery
ACEI: Angiotensin converting enzyme inhibitors
ARB: Angiotensin II receptor blocker
ARNI: Angiotensin receptor enkephalinase inhibitor
HDL-C: High-density lipoprotein cholesterol
LDL-C: Low-density lipoprotein cholesterol

## References

[1] Reynolds HR, Picard MH, Spertus JA, Peteiro J, Lopez Sendon JL, Senior R, El-Hajjar MC, Celutkiene J, Shapiro MD, Pellikka PA, Kunichoff DF, Anthopolos R, Alfakih K, Abdul-Nour K, Khouri M, Bershtein L, De Belder M, Poh KK, Beltrame JF, Min JK, Fleg JL, Li Y, Maron DJ, Hochman JS (2021). Natural history of patients with ischemia and no obstructive coronary artery disease: The CIAO-ISCHEMIA study. Circulation.

[2] Beltrame JF (2012). Defining the coronary slow flow phenomenon. Circ J.

[3] Saadat M, Masoudkabir F, Afarideh M, Ghodsi S, Vasheghani-Farahani A (2019). Discrimination between obstructive coronary artery disease and cardiac syndrome X in women with typical angina and positive exercise test; utility of cardiovascular risk calculators. Medicina (Kaunas).

[4] Wang Y, Jia PY, Chen BJ, Chen Y, Yu H, Yu Y, Yang HM, Jia DL, Ma CY (2017). Evaluation of plasma thrombomodulin in patients with coronary slow flow. Cardiology.

[5] Guerrero-Romero F, Simental-Mendía LE, González-Ortiz M, Martínez-Abundis E, Ramos-Zavala MG, Hernández-González SO, Jacques-Camarena O, Rodríguez-Morán M (2010). The product of triglycerides and glucose, a simple measure of insulin sensitivity. Comparison with the euglycemic-hyperinsulinemic clamp. J Clin Endocrinol Metab.

[6] Won KB, Park GM, Lee SE, Cho IJ, Kim HC, Lee BK, Chang HJ (2018). Relationship of insulin resistance estimated by triglyceride glucose index to arterial stiffness. Lipids Health Dis.

[7] Boden G (2011). Obesity, insulin resistance and free fatty acids. Curr Opin Endocrinol Diabetes Obes.

[8] Karczewski J, Śledzińska E, Baturo A, Jończyk I, Maleszko A, Samborski P, Begier-Krasińska B, Dobrowolska A (2018). Obesity and inflammation. Eur Cytokine Netw.

[9] Nordestgaard BG (2016). Triglyceride-rich lipoproteins and atherosclerotic cardiovascular disease: new insights from epidemiology, genetics, and biology. Circ Res.

[10] Er LK, Wu S, Chou HH, Hsu LA, Teng MS, Sun YC, Ko YL (2016). Triglyceride glucose-body mass index is a simple and clinically useful surrogate marker for insulin resistance in nondiabetic individuals. PLoS ONE.

[11] Dai XT, Kong TZ, Zhang XJ, Luan B, Wang Y, Hou AJ (2022). Relationship between increased systemic immune-inflammation index and coronary slow flow phenomenon. BMC Cardiovasc Disord.

[12] Ozcan T, Gen R, Akbay E, Horoz M, Akcay B, Genctoy G, Muslu N, Camsari A, Cicek D, Gok E, Kiykim A (2008). The correlation of thrombolysis in myocardial infarction frame count with insulin resistance in patients with slow coronary flow. Coron Artery Dis.

[13] Genc Tapar G, Elcik D, Dogan A, Altunel E, Inanc MT, Alcali B, Boylug S, Oguzhan A, Topsakal R, Ergin A, Kalay N (2020). An investigation of the relationship between arterial aortic stiffness and coronary slow flow that was detected during coronary angiography. Echocardiography.

[14] Gibson CM, Cannon CP, Daley WL, Dodge JT, Alexander B, Marble SJ, McCabe CH, Raymond L, Fortin T, Poole WK, Braunwald E (1996). TIMI frame count: a quantitative method of assessing coronary artery flow. Circulation.

[15] Beltrame JF, Limaye SB, Horowitz JD (2002). The coronary slow flow phenomenon: a new coronary microvascular disorder. Cardiology.

[16] Goel PK, Gupta SK, Agarwal A, Kapoor A (2001). Slow coronary flow: a distinct angiographic subgroup in syndrome X. Angiology.

[17] Yesin M, Çağdaş M, Karabağ Y, Rencüzoğullari İ, Burak C, Kalçik M, Gürsoy MO, Karakoyun S (2019). Assessment of the relationship between C-reactive protein-to-albumin ratio and slow coronary flow in patients with stable angina pectoris. Coron Artery Dis.

[18] Camsari A, Ozcan T, Ozer C, Akcay B (2008). Carotid artery intima-media thickness correlates with intravascular ultrasound parameters in patients with slow coronary flow. Atherosclerosis.

[19] Cin VG, Pekdemir H, Camsar A, Ciçek D, Akkus MN, Parmaksýz T, Katýrcýbaý T, Döven O (2003). Diffuse intimal thickening of coronary arteries in slow coronary flow. Jpn Heart J.

[20] Tao LC, Xu JN, Wang TT, Hua F, Li JJ (2022). Triglyceride-glucose index as a marker in cardiovascular diseases: landscape and limitations. Cardiovasc Diabetol.

[21] Yang J, Tang YD, Zheng Y, Li C, Zhou Q, Gao J, Meng X, Zhang K, Wang W, Shao C (2021). The impact of the triglyceride-glucose index on poor prognosis in nondiabetic patients undergoing percutaneous coronary intervention. Front Endocrinol.

[22] Wang Y, Wang Y, Sun S, Liu X, Zhao W, Li W, Suo M, Wu Z, Wu X (2022). Triglyceride-glucose index level and variability and outcomes in patients with acute coronary syndrome undergoing percutaneous coronary intervention: an observational cohort study. Lipids Health Dis.

[23] Wang X, Xu W, Song Q, Zhao Z, Meng X, Xia C, Xie Y, Yang C, Jin P, Wang F (2022). Association between the triglyceride-glucose index and severity of coronary artery disease. Cardiovasc Diabetol.

[24] Chen S, Mei Q, Guo L, Yang X, Luo W, Qu X, Li X, Zhou B, Chen K, Zeng C (2022). Association between triglyceride-glucose index and atrial fibrillation: A retrospective observational study. Front Endocrinol (Lausanne).

[25] Guo X, Shen R, Yan S, Su Y, Ma L (2023). Triglyceride-glucose index for predicting repeat revascularization and in-stent restenosis in patients with chronic coronary syndrome undergoing percutaneous coronary intervention. Cardiovasc Diabetol.

[26] Yan Y, Wang D, Sun Y, Ma Q, Wang K, Liao Y, Chen C, Jia H, Chu C, Zheng W, Hu J, Yuan Y, Wang Y, Wu Y, Mu J (2022). Triglyceride-glucose index trajectory and arterial stiffness: results from Hanzhong Adolescent Hypertension Cohort Study. Cardiovasc Diabetol.

[27] Wang M, Mei L, Jin A, Cai X, Jing J, Wang S, Meng X, Li S, Wei T, Wang Y, Pan Y (2022). Association between triglyceride glucose index and atherosclerotic plaques and Burden: findings from a community-based study. Cardiovasc Diabetol.

[28] Li W, Chen D, Tao Y, Lu Z, Wang D (2022). Association between triglyceride-glucose index and carotid atherosclerosis detected by ultrasonography. Cardiovasc Diabetol.

[29] Yuksel Y, Yildiz C (2023). Evaluation of triglyceride-glucose index in coronary slow flow patients. Kardiologiia.

[30] Molina MN, Ferder L, Manucha W (2016). Emerging role of nitric oxide and heat shock proteins in insulin resistance. Curr Hypertens Rep.

[31] Gerrits AJ, Koekman CA, van Haeften TW, Akkerman JW (2010). Platelet tissue factor synthesis in type 2 diabetic patients is resistant to inhibition by insulin. Diabetes.

[32] Cheng Y, Fang Z, Zhang X, Wen Y, Lu J, He S, Xu B (2023). Association between triglyceride glucose-body mass index and cardiovascular outcomes in patients undergoing percutaneous coronary intervention: a retrospective study. Cardiovasc Diabetol.

[33] Hawkins BM, Stavrakis S, Rousan TA, Abu-Fadel M, Schechter E (2012). Coronary slow flow- prevalence and clinical correlations. Circ J.

